# Parasite responses to resource provisioning can be altered by within-host co-infection interactions

**DOI:** 10.1098/rspb.2025.1840

**Published:** 2025-11-05

**Authors:** Diana Erazo, Amy R. Sweeny, Amy B. Pedersen, Andy Fenton

**Affiliations:** ^1^ Institute of Infection, Veterinary, and Ecological Sciences, University of Liverpool, Liverpool, UK; ^2^ Department of Biology, Royal Museum for Central Africa, Tervuren, Flanders, Belgium; ^3^ Evolutionary Ecology Group (EVECO), University of Antwerp, Antwerp, Flanders, Belgium; ^4^ Spatial Epidemiology Laboratory (SpELL), Université Libre de Bruxelles, Brussels, Belgium; ^5^ Institute of Ecology and Evolution, University of Edinburgh, Edinburgh, UK

**Keywords:** resource provisioning, co-infection, host–parasite interactions, mathematical model, parasite transmission

## Abstract

Anthropogenic changes to the environment significantly impact wildlife infectious diseases by modifying food resources and impacting host–parasite interactions through changes in host demography, behaviour and immune defences. Supplemental resource provisioning has been found to both enhance and mitigate parasite transmission; however, the role of co-infecting parasites in mediating these effects remains understudied. We developed a mathematical model to explore these dynamics, motivated by the empirical system of wood mice (*Apodemus sylvaticus*) infected with the nematode *Heligmosomoides polygyrus*, which suppresses co-infections by the apicomplexan microparasite *Eimeria hungaryensis*. Our model shows that the effects of resource provisioning on parasite epidemiology can be mediated, and potentially reversed, by within-host co-infection interactions, through effects on host–parasite contact rates and host susceptibility. Provisioning may elevate microparasite prevalence by reducing nematode burdens, thereby releasing the microparasite from the negative effects of co-infection. However, if provisioning increases host contact rates with parasite infective stages in the environment, the associated increase in nematode burdens can result in concomitant reductions in the microparasite, owing to the negative within-host co-infection interaction. Our study highlights the need for experimental designs that decouple the complex factors of provisioning on co-infecting parasite dynamics and provides a framework for interpreting outcomes in multi-parasite systems.

## 1. Introduction

Anthropogenic changes to the environment, such as urbanization, agricultural expansion, infrastructure development and land-use change, have the potential to impact the occurrence and spread of infectious diseases in wildlife populations [1,2]. In particular, anthropogenic changes can alter the abundance, distribution and quality of food resources in the environment, which can significantly affect the spread and impact of infectious diseases in wildlife [3]. Empirical studies support three mechanisms by which resource provisioning can impact host–parasite interactions: changes in host demography, behaviour and immune defences [4,5]. For example, when food availability is not limited because of changes in access, animal population densities can increase and host vital rates can improve, leading to higher parasite prevalence, as has been documented in bumblebees in urban versus rural gardens [6]. Increased supplementation can also increase host aggregation and consequently contact rates around resource patches, potentially facilitating transmission [7]. Such behavioural changes have been observed in house finches, which have increased flocking around bird feeders, leading to increases in the prevalence of *Mycoplasma gallisepticum,* which causes conjunctivitis [8]. By contrast, both host condition and immune defences may improve under resource provisioning, leading to a decrease in parasite infection. For instance, the nutritional stress, body condition and immune function of kit foxes was found to be enhanced in urban settings where food and water were copious [9]. Importantly, resource provisioning can impact these processes simultaneously, resulting in complex outcomes for parasite dynamics [4,5,10]. Therefore, observed epidemiological outcomes to provisioning occur as a balance among different mechanisms acting at the within-host, between-host and population scales, each of which may be affected differently by provisioning [4,5,10].

The effects of resource provisioning have been studied theoretically and empirically across a diverse range of host–parasite systems varying in parasite transmission modes and host and parasite life histories [4,5,10]. However, studies to date have focused exclusively on one-host–one-parasite systems. As such, this work excludes effects of provisioning mediated by interactions among co-infecting parasites. Co-infection, or the simultaneous infection of hosts by multiple parasite species, is standard in natural systems, and there can be powerful within-host interactions between co-infecting parasites that alter host susceptibility, parasite burdens and disease severity of the parasites [11–13]. Co-infections between macroparasites (such as helminths and ectoparasites) and microparasites (such as viruses, bacteria and protozoa) have been highlighted as particularly important, owing to the ubiquity of helminth (worm) infections in humans and animals and the strong immunomodulatory consequences that worms can have on their hosts [14–16], with potentially significant effects on host susceptibility to microparasites [11,17–20]. Given these potentially strong within-host interactions, we may expect the effects of resource provisioning on one parasite to be mediated by the possible differential effects of provisioning on other, co-infecting parasites. However, despite recent advances in understanding how resource provisioning may affect disease dynamics in wildlife, how interactions between co-infecting parasites shape those impacts has yet to be explored. In this study, we investigate how within-host interactions between co-infecting parasites mediate the effects of resource provisioning on infection dynamics. We develop a generalized theoretical framework to explore the consequences of differential responses to provisioning among co-infecting parasites, ground-truthed with reference to an empirical system, where previous experiments have demonstrated strong interactive effects between a co-infecting helminth and microparasite: wild wood mice co-infected with a nematode macroparasite and protozoan microparasites. Through this combined approach, we seek to determine how within-host co-infection interactions impact the response of resource provisioning on disease dynamics.

Wood mice (*Apodemus sylvaticus*) in the UK are commonly infected by the gastrointestinal nematode (*Heligmosomoides polygyrus*) and several species of apicomplexan microparasites belonging to the genus *Eimeria* [21,22]. Previous work on this system has shown that *H. polygyrus* co-infection has a strong effect on one species of *Eimeria* (*Eimeria hungaryensis*) sharing a common infection site within the host. Treatment with the anti-nematode drug Ivermectin reduced *H. polygyrus* prevalence by 70% and led to 15-fold increase in the shedding of *E. hungaryensis* oocysts compared with control (untreated) individuals [22]. In addition, results from controlled co-infection experiments using wild-collected isolates of *H. polygyrus* and *E. hungaryensis* in a captive, wild-derived wood mouse colony closely match those from the field experiment, showing that co-infection with *H. polygyrus* significantly reduces both peak and total shedding of *E. hungaryensis* oocysts [21]. We hypothesize that the effect of *H. polygyrus* infection was particularly strong on *E. hungaryensis* because both parasites share a common infection site in the duodenum. However, other *Eimeria* species (e.g. *Eimeria uptoni*), which are found lower in the small intestine and large intestine, did not significantly increase after anthelmintic treatment [22]. These results clearly show that *H. polygyrus* suppresses *E. hungaryensis* transmission potential in co-infected wood mice and provide a fascinating case for studying the role of provisioning on co-infection dynamics. Moreover, recent experimental work shows that supplemental nutrition in wood mice can reduce helminth burdens and improve the efficacy of antiparasitic treatment, highlighting the relevance of provisioning-induced changes in host condition and immune function for infection outcomes [23]. The wood mouse system, therefore, provides an ideal empirical case study to inform development of a mathematical model to investigate theoretically how microparasite–macroparasite co-infection interactions moderate the effects of resource provisioning on parasite dynamics.

## 2. Methods

We constructed a microparasite–macroparasite co-infection model reflecting the co-infection interaction dynamics of *Eimeria* sp.–*H. polygyrus* in wood mice. To ensure realistic parameter ranges were considered, demographic and disease-related parameters were estimated using previous longitudinal data on uniquely passive integrated transponder tagged mice captured in a 4 year experiment where infection and burdens were measured across the lifespan of mice [22,24,25]. With the parameterized model, we explored the impact of different hypothetical provisioning scenarios on long-term mean parasite infection levels, following the approach of Becker & Hall [4].

### (a) Model

**Figure 1.**
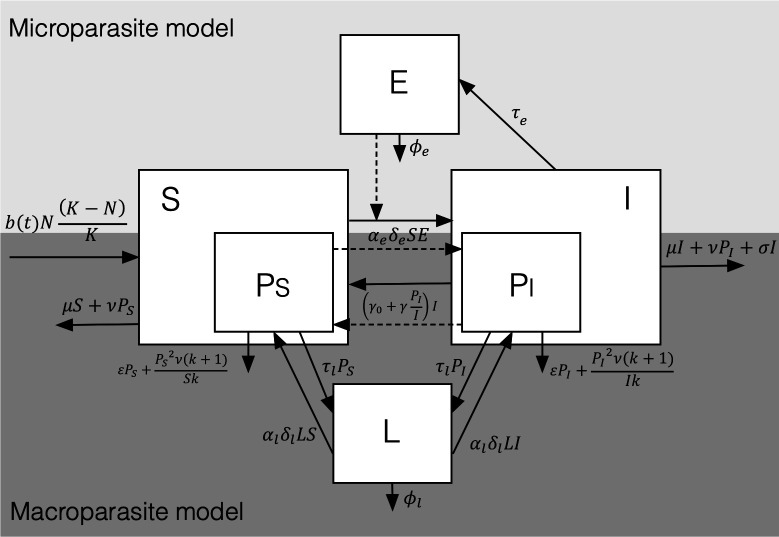
Schematic diagram of the microparasite–macroparasite co-infection model. The light grey area illustrates the portion that describes microparasite transmission, with susceptible (S) and infected (I) hosts interacting with environmental infective stages (E). The dark grey area depicts the model portion that describes macroparasite transmission, tracking total worm burdens in susceptible and infected hosts ($P_{S}$, $P_{I}$) and environmental stages (L). Solid arrows indicate processes such as infection, recovery, shedding and mortality. Dashed arrows denote encounters between E and S, and the movement between $P_{S}$ and $P_{I}$ owing to microparasite infection or recovery. Parameters include host–parasite encounter rates ($\alpha_{e},\alpha_{l}$), host susceptibilities ($\delta_{e},\delta_{l}$), parasite shedding rates ($\tau_{e},\tau_{l}$), parasite mortality rates (of adult macroparasites: ε and environmental stages: $\phi_{e},\phi_{l}),$ host mortality rates (background: μ; macroparasite-induced: ν and microparasite-induced: σ), host recovery rate from microparasite infection ($\gamma_{0},\;\gamma$), macroparasite aggregation (k), host birth rate (b(t)) and host-carrying capacity (*K*).

Our co-infection model is based on the microparasite–macroparasite model framework developed by Fenton [17], which considers a hybrid of the two separate models described by Anderson & May [26–28] (figure 1).

The microparasite model describes the dynamics of an environmentally transmitted susceptible–infected–susceptible microparasite (e.g. *Eimeria* sp. in the wood mouse system, an apicomplexan that undergoes multiple cycles of replication within the host). Host population dynamics are defined by two variables, S and I describing the densities of microparasite-susceptible (uninfected) and microparasite-infected animals, respectively. Hosts are assumed to get infected by the microparasite through ingestion of infective stages (e.g. *Eimeria* sp. oocysts) in the environment (E) at rate $\alpha_{e}\delta_{e}SE$, where $\alpha_{e}$ is the *per capita* encounter rate between susceptible hosts (*S*) and microparasite infective stages (*E*), and $\delta_{e}$ is host susceptibility to the microparasite (the probability that the encounter results in infection). Each infected individual sheds microparasite infective stages at rate $\tau_{e}$ and recovers (back to susceptible mice) at baseline rate $\gamma_{0}$; this recovery rate may be affected by macroparasite co-infection (see later). Microparasite infective stages in the environment were assumed to lose the ability to infect at rate $\phi_{e}$.

In contrast to the microparasite model, the macroparasite model keeps track of the total numbers of macroparasites (total worm burdens, $P_{S},P_{I}$) within hosts of classes S and I, respectively (hence the mean worm burdens per class are PSS and PII, respectively). These macroparasites release free-living infective stages (L) at *per capita* rate $\tau_{l}$ (assumed to be the same for macroparasites in both host compartments S and I), which die at rate $\phi_{l}$. Adult macroparasites $(P_{S},P_{I}$) are acquired when hosts (S,I) encounter these infective stages in the environment, which occurs at rate $\alpha_{l}\delta_{l}HL$, where H is the relevant host compartment (H∈{S,I}), $\alpha_{l}$ is the *per capita* host encounter rate with macroparasite infective stages (*L*) and $\delta_{l}$ is host susceptibility to the macroparasite (the probability that the encounter results in infection). The macroparasite population in each host compartment may decrease by multiple factors: host natural mortality (μ), macroparasite natural mortality (ε) and host disease-induced mortalities (ν and σ). Burden-dependent host mortality owing to the macroparasite is modelled as $\frac{{P_{S}}^{2}\nu (k+1)}{Sk}$ and $\frac{{P_{I}}^{2}\nu (k+1)}{Ik}$ for susceptible and infected hosts respectively, following the formulation in [17] (appendix C), which derives this expression from the second moment of a negative binomial distribution. This structure captures mortality that scales with parasite burden and incorporates the degree of parasite aggregation (k).

In the wood mouse system, we previously found evidence of a negative within-host interaction between the macroparasite *H. polygyrus* and the microparasite *E. hungaryensis*, since anthelmintic treatment resulted in increased abundance of *E. hungaryensis* [22]; however, no effect of antiparasite treatment was seen for *E. uptoni*. To incorporate the possibility of such a co-infection interaction, an additional term $\left(\gamma \frac{P_{I}}{I}\right)$, reflecting a potential increase in host recovery from the microparasite owing to the effect of the macroparasite, was included in the model, where γ represents the extra recovery rate from microparasite infection for co-infected hosts, dependent on the mean burden of macroparasite infection $\left(\frac{P_{I}}{I}\right)$. The outcomes of models including this interaction term were compared with the cases where the macroparasite and microparasite infect independently and do not interact (e.g. as seen for other *Eimeria* species that do not compete with the nematode, such as *E. uptoni*); in those cases γ=0, removing this interaction term. Note, we focus on a unidirectional co-infection interaction of the macroparasite on the microparasite (reflecting the observed effect of *H. polygyrus* on *E. hungaryensis*, as the dominant co-infection interaction detected in system). Our previous controlled co-infection experiments conducted in our laboratory wood mouse colony have shown that *Eimeria* co-infection does impact the duration and magnitude of *H. polygyrus* egg shedding [21]. However, incorporating bidirectional co-infection interactions would greatly complicate the model analysis and interpretation, so here we focus solely on the impact of the microparasite on the microparasite, but we consider the implications of incorporating this aspect in the Discussion.

To help ground-truth the model and reflect host dynamics in the wood mouse system, susceptible hosts were assumed to increase at a seasonally varying rate $b(t)N\frac{\left(K-N\right)}{K}$, where K is the carrying capacity, b(t) the birth rate at time *t* (see below) and N is the total host population density (S+I). To capture seasonal dynamics, we defined birth rate as a function of time, as follows:


$$b\left(t\right)=\left|b_{0}\;sin\;2\pi \left(\frac{t}{52}-\omega \right)\right|+b_{0}\;sin\;2\pi \left(\frac{t}{52}-\omega \right),$$

where t is time in weeks, $b_{0}$ is the baseline birth rate and ω is the birth function phase (which sets the timing of peak births). This construction means b(t) is periodic with a period of 1 year, allowing for a six month-long breeding season and six month-long period with no reproduction, reflecting breeding patterns seen in wood mice in the UK [29].

The full co-infection model is, therefore, as follows:


(2.1) $$\frac{dS}{dt}=\;b\left(t\right)N\frac{\left(K-N\right)}{K}-\alpha_{e}\delta_{e}SE+\left(\gamma_{0}+\gamma \frac{P_{I}}{I}\right)I-\mu S-\nu P_{S},$$

(2.2) $$\frac{dI}{dt}=\;\alpha_{e}\delta_{e}SE-\left(\gamma_{0}+\gamma \frac{P_{I}}{I}\right)I-\mu I-\nu P_{I}-\sigma I,$$

(2.3) $$\frac{dE}{dt}=\;\tau_{e}I-\phi_{e}E,$$

(2.4) $$\frac{dP_{S}}{dt}=\;\alpha_{l}\delta_{l}LS\;+\left(\gamma_{0}+\gamma \frac{P_{I}}{I}\right)P_{I}-\left(\mu +\nu +\varepsilon \right)P_{S}-\alpha_{e}\delta_{e}ES\frac{P_{S}}{S}-\frac{P_{S}^{2}\nu \left(k+1\right)}{Sk},$$

(2.5) $$\frac{dP_{I}}{dt}=\;\alpha_{l}\delta_{l}LI-\left(\gamma_{0}+\gamma \frac{P_{I}}{I}\right)P_{I}-\left(\mu +\nu +\varepsilon +\sigma \right)P_{I}+\alpha_{e}\delta_{e}ES\frac{P_{S}}{S}-\frac{P_{I}^{2}\nu \left(k+1\right)}{Ik},$$

(2.6) $$\frac{dL}{dt}=\;\tau_{l}P_{I}+\tau_{l}P_{S}-\phi_{l}L.$$

Note that if infected hosts (I) recover from the microparasite, the corresponding $P_{I}$ population transfers to $P_{S}$ through the term $\left(\gamma_{0}+\gamma \frac{P_{I}}{I}\right)P_{I}$. Whereas when susceptible hosts (S) get infected with the microparasite, an average$\frac{P_{S}}{S}$ worms transfer to $P_{I}$ through the term $\alpha_{e}\delta_{e}ES\frac{P_{S}}{S}$.

### (b) Model fitting and parameter estimation

Description of the steps taken to obtain estimates of parameters related to wood mice and parasite infective stage dynamics can be found in the electronic supplementary material. Briefly, infection-related parameters for the microparasite (*Eimeria* spp.) and macroparasite (*H. polygyrus*) $\left(\xi =\left\{\beta_{e},\;\;\beta_{l},\;\gamma_{0},\;\gamma ,\;\nu ,\sigma \right\}\right)$ were estimated by fitting the co-infection model to weekly wood mouse trapping data, collected between June 2009 and December 2012 (see the electronic supplementary material for details of data collection). Note that the transmission rate for both parasites is the product of contact rate and susceptibility (*E. hungaryensis:* $\alpha_{e}\delta_{e}$ and *H. polygyrus:* $\alpha_{l}\delta_{l}$); thus, for model fitting, each of these products were considered as one single parameter for each parasite (*E. hungaryensis:* $\alpha_{e}\delta_{e}=\beta_{e}$ and *H. polygyrus:* $\alpha_{l}\delta_{l}$ = $\beta_{l}$). For the subsequent provisioning analysis (see below), these transmission term parameters were treated separately as stated in the model construction because resource provisioning may induce different responses in direction and magnitude for contact rate and susceptibility. The partitioning of the *β* terms into susceptibility and contact parameters was done following the logic presented in [10].

The infection-related parameters were estimated using adaptive Monte Carlo Markov chain Metropolis-Hastings (MCMC-MH), assuming uniform priors (table 1A), using the R package fitR (v. 0.2.1). The first 10 years (530 weeks) of predicted transient dynamics of the simulation were ignored as burn-in time, and the subsequent 4 years of simulation were fitted to 4 years of data. The weekly predicted number of microparasite-infected mice and total number of macroparasite infective stages released by mice were fitted to the observed numbers of *E. hungaryensis* infected mice and total *H. polygyrus* egg counts in faecal samples per week. The observed number of microparasite-infected mice was assumed to follow a Poisson distribution, and the number of macroparasite infective stages was assumed to follow a negative binomial distribution. The overall log-likelihood of the data is then given by the sum of the log-likelihoods of seeing the observed number of *E. hungaryensis*-infected hosts ($l_{Eimeria\_inf-\text{week}}\left(y_{\text{week}}|\xi \right)$) and the observed number of *H. polygyrus* infective stages ($l_{Hpoly\_EPG-\text{week}}\left(y_{\text{week}}|\xi \right)$) each week, given the parameter values ξ:

**Table 1.** Infection-related parameter names and values estimated from fitting to data from the UK wood mouse system. (A) Baseline epidemiological parameters; estimated values show median and 95% credible intervals from the posterior distributions of each parameter, and the values used for the provisioning analyses. (B) Baseline, minimum and maximum values of parameters for the provisioning analysis.

A					
symbol	parameter description	initial value for model fitting	prior distribution	estimated value	value used in provisioning analysis
** $\beta_{l}$ **	macroparasite (e.g. *H. polygyrus*) transmission rate	$1x10^{-6}$	0-0.1	$5.628\times 10^{-6}$ $\left[5.619\times 10^{-6}-5.637\times 10^{-6}\right]$	$5.6\times 10^{-6}$
** $\beta_{e}$ **	microparasite (e.g. *Eimeria* spp.) transmission rate	$1x10^{-5}$	0-0.1	$2.612\times 10^{-6}$ $\left[2.603\times 10^{-6}-2.621\times 10^{-6}\right]$	$2.6\times 10^{-6}$
** $\gamma_{0}$ **	baseline microparasite (*Eimeria* spp.) recovery rate	0.01	λ=2.8	0.389 [0.385-0.392]	0.39
** γ **	additional microparasite (e.g. *E. hungaryensis*) recovery rate owing to co-infection	0.1	λ=10	0.481 [0.476-0.485]	0.48
** ν **	macroparasite *per capita* disease-induced host mortality rate	0.1	0-1	0.00295 [0.00292-0.00298]	0.003
** σ **	microparasite *per capita* disease-induced host mortality rate	0.1	0-1	0.0118 [0.0116-0.0120]	0.01

B					
symbol	parameter description	baseline value	minimum value	maximum value	
$\alpha =\alpha_{l}=\alpha_{e}$	contact rate	$1\times 10^{-5}$	$1\times 10^{-5}$	$2\times 10^{-5}$	
$\delta_{l}$	*H. polygyrus* susceptibility	0.56	0.28	0.56	
$\delta_{e}$	*E. hungaryensis* susceptibility	0.26	0.13	0.26	


$$l_{all}\;\left(\text{data}|\xi \right)=\sum_{\text{week}}l_{Eimeria\_inf-\text{week}}\left(y_{\text{week}}|\xi \right)+\sum_{\text{week}}l_{Hpoly\_EPG-\text{week}}\left(y_{\text{week}}|\xi \right).$$

Four MCMC-MH chains of 10 000 iterations were run using the default parameter standard deviation (parameter value divided by 10). Then, for each chain, using the first chain output (standard deviation and $\bar{\xi }$ for the last 9000 iterations) as input, we ran a second chain of 1 00 000 iterations. For the second chain, the first 5000 iterations were discarded (burn-in), and we eliminated every 10 samples per sample to avoid auto-correlation (thinning). The Gelman–Rubin diagnostic was used to assess MCMC convergence by analysing the difference between chains. For the provisioning analyses (below), we used rounded values of the estimated parameters and ensured all model solutions were biologically feasible (non-negative) under all provisioning scenarios (see table 1A).

### (c) Resource provisioning analysis

**Figure 2.**
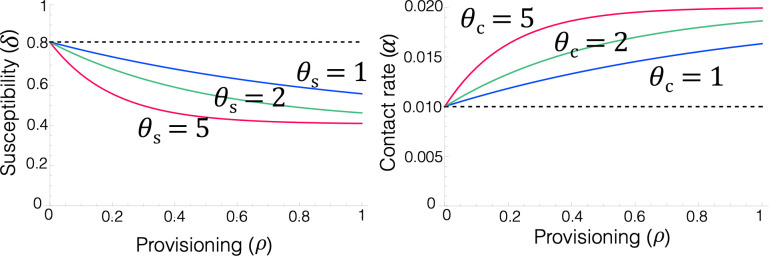
Illustration of how resource provisioning was incorporated into the model and how we quantified their epidemiological effects. Functional forms used to represent the impact of provisioning (ρ, *x*-axis) on host susceptibility (δ; decreasing) and contact rate (α; increasing). Coloured lines represent different values of the sensitivity parameters ($\theta_{s}$ for susceptibility and $\theta_{c}$ for contact rate), determining how strongly each trait responds to provisioning. The dashed horizontal line indicates the baseline level (i.e. no provisioning, ρ=0). Adapted from [10].

Our analysis of the impacts of provisioning followed the approach of Becker & Hall [5] in which the parameter ρ denotes the extent of provisioning, such that increasing values represented increased resource availability [5]. Specifically, ρ takes values between 0 and 1, thus ρ=0 means no provisioning (baseline parameter values) and ρ=1 corresponds to arbitrarily high levels of provisioning. We assumed that in the short-term, resource provisioning could affect host *per capita* contact rates with the environmental pools of parasite infective stages ($\alpha_{e},\alpha_{l})$ and/or host susceptibility ($\delta_{e},\delta_{l})$, probability of infection after exposure, for both parasites. For simplicity, we ignored host demographic responses owing to provisioning as we assumed they occur over longer timescales than we consider here. We also did not include potential effects of provisioning on host ‘tolerance’ (the ability of hosts to withstand potentially high infection levels; [30,31]), ‘resistance’ (the fitness or survival costs of reducing parasite burdens; [32]) or host immune effects on within-host parasite reproduction or survival. We also did not consider possible effects of provisioning on parasite traits, such as shedding or survival rates of parasite infective stages in the environment through increased resource levels acquired within the host. We consider the implications of some of these aspects more fully in the Discussion. As done in previous provisioning analyses [5,10], the response of each parameter to provisioning was assumed to be monotonic and saturating, either increasing or decreasing, at a rate dependent on parameter sensitivity to provisioning ($\theta_{x}$); these $\theta_{x}$ values allow parameters to scale from linear forms ($\theta_{x}$ = 1) to a quickly saturating shape ($\theta_{x}$ >>1) (see figure 2 for illustrative examples of these relationships).

Theoretical and empirical research indicate that provisioning can boost host contact rates, owing to hosts aggregating around food sources, thereby elevating pathogen prevalence and outbreak intensity [33,34]. Therefore, we assumed contact rates ($\alpha_{e},\alpha_{l})$ increased with provisioning according to the functional form:


$$\alpha =\alpha_{\text{max}}-\left(\alpha_{\text{max}}-\alpha_{\text{min}}\right)e^{-\theta_{c}\rho },$$

where $\alpha_{\text{min}}$ and $\alpha_{\text{max}}$ are the minimum and maximum values that the contact rate parameters could take, respectively. Conversely, other studies indicate that when food quality is high and nutritionally balanced, provisioning can enhance host nutrition and immune defence, leading to a reduction in infection burdens [1,35,36]. As a result, we assumed host susceptibility ($\delta_{e},\delta_{l})$ decreased with provisioning according to the following function:


$$\delta_{l}=\delta_{l\text{min}}+\left(\delta_{l\text{max}}-\delta_{l\text{min}}\right)e^{-\theta_{l}\rho },$$

$$\delta_{e}=\delta_{e\text{min}}+\left(\delta_{e\text{max}}-\delta_{e\text{min}}\right)e^{-\theta_{e}\rho },$$

where $\delta_{lmin}$ and $\delta_{emin}$ are the minimum values and $\delta_{lmax}$ and $\delta_{emax}$ are the maximum values that those parameters could take. We assumed the baseline estimated values represented no provisioning (ρ=0). We then arbitrarily assumed that in a high provisioning scenario (ρ=1), the maximum contact rate is twice the baseline value ($\alpha_{\text{max}}=2\alpha_{\text{min}/bl}$) and the minimum susceptibility is half the baseline value ($\delta_{e\text{min}}=\frac{\delta_{e\text{max}/bl}}{2},\;\delta_{l\text{min}}=\;\frac{\delta_{l\text{min}/bl}}{2}$) (table 1B).

The net effect of resource provisioning in the co-infection system was investigated by calculating long-term macroparasite mean burdens, and prevalences of the ‘interacting microparasite’ (e.g. *E. hungaryensis* in the UK wood mouse system) and the ‘non-interacting microparasite’ (e.g. *E. uptoni*, which was assumed to have the same life-history parameters as *E. hungaryensis* but not affected by nematode co-infection; *γ* = 0). Long-term mean values for each of these parasites were obtained numerically by running model simulations for 20 years and averaging the last 10 years. To assess the impact of resource provisioning, we calculated the difference in these long-term mean values between high provisioning (ρ=1) and no provisioning (ρ=0) for different combinations of ‘provisioning sensitivity parameters’ $\theta_{c},\theta_{l}$ and $\theta_{e}$. To simplify the analyses and aid clarity of presentation, we expressed $\theta_{c}$ (the sensitivity of contact rates to provisioning) and $\theta_{l}$ (the sensitivity of macroparasite susceptibility to provisioning) relative to $\theta_{e}$ (the sensitivity of microparasite susceptibility to provisioning; i.e. $\Theta_{c}$=$\theta_{c}$/$\theta_{e}$ and $\Theta_{l}$=$\theta_{l}$/$\theta_{e}$), and arbitrarily set $\theta_{e}$ to a value of 1 (again, the various provisioning-related parameters have little direct connection to measurable properties of the natural system, so we focus primarily on the relative effects of these different parameters). Hence, values of $\Theta_{c}$ (or $\Theta_{l}$) that are greater than 1 imply that contact rate (or susceptibility to the macroparasite) is more sensitive to the effects of provisioning than susceptibility to the microparasite; conversely values of $\Theta_{c}$ (or $\Theta_{l}$) < 1 imply that microparasite susceptibility is more sensitive to provisioning than contact rate (or macroparasite susceptibility). We then simultaneously varied both $\Theta_{c}$ and $\Theta_{l}$ systematically from 0 to 5 and, for all combinations assessed the changes in long-term mean prevalence of the interacting and non-interacting microparasites, and long-term macroparasite mean burden, under high provisioning (ρ=1) relative to no provisioning (ρ=0).

## 3. Results

### (a) Model fitting

**Figure 3.**
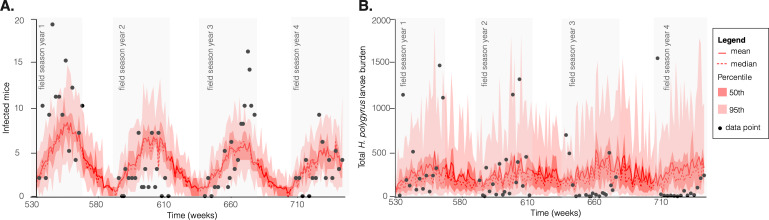
Microparasite–macroparasite model fit to data from UK wood mice. Panels show model fit to (A) number of *E. hungaryensis*-infected mice and (B) mean *H. polygyrus* infective stage shedding rate per mouse, over time (weeks). The black dots depict data from the wood mouse system, and grey shaded regions show the periods in which mice were trapped and data collected. Model simulation mean and median are shown by the red and red dashed line, respectively. Pink and red shades represent 50th and 95th percentile of the simulations, respectively.

Model fitting to *E. hungaryensis* infection and *H. polygyrus* mean burden data is shown in figure 3. The transmission rate of *Heligmosomoides* $\left(\alpha_{l}\delta_{l}\;=\;\beta_{l}=5.63\times 10^{-6}\;\left[5.62\times 10^{-6}-5.64\times 10^{-6}\right]\right)$ (table 1A) was estimated to be higher than the transmission rate of *E. hungaryensis* $\left(\alpha_{e}\delta_{e}\;=\;\beta_{e}=2.61\times 10^{-6}\;\left[2.60\times 10^{-6}-2.62\times 10^{-6}\right]\right)$. We estimated that the baseline mean recovery time from *E. hungaryensis* infection was under three weeks $\left(\gamma_{0}=0.389\;\left[0.385-0.392\right]\;week^{-1}\right)$ and, when coinfected with *H. polygyrus*, recovery time decreased by half a week $\left(\gamma =0.481\;\left[0.476-0.485\right]\;week^{-1}\right)$. *Heligmosomoides polygyrus* disease-induced mortality in wild mice was estimated to be $2.95\times 10^{-3}\;\left[2.92\times 10^{-3}-2.98\times 10^{-3}\right]$ mice worm^−1^ week^−1^, and *E. hungaryensis* disease-induced mortality was 0.0118 [0.0116−0.0120] mice week^−1^. It is important to note that the 95% credible intervals shown in table 1 reflect uncertainty in the estimated parameter values from the posterior distributions, while the shaded regions in figure 3 represent the variability in predicted model outputs. As such, individual data points may fall outside the prediction envelope even when parameter estimates are well constrained.

### (b) Provisioning analysis

**Figure 4.**
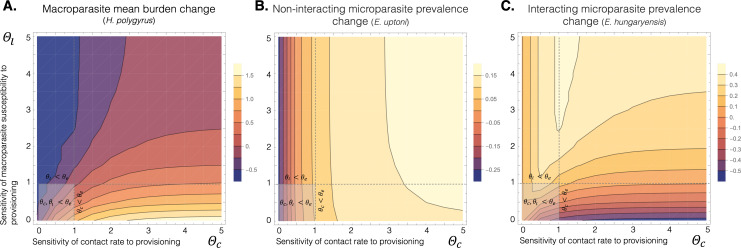
Predicted microparasite and macroparasite outcomes owing to resource provisioning. (A) Macroparasite (e.g. *H. polygyrus*) mean burden change owing to provisioning (*ρ* = 1), relative to the predicted mean burden in the absence of provisioning (*ρ* = 0). (B) Change in long-term mean prevalence of the ‘non-interacting’ microparasite (e.g. *E. uptoni*) owing to provisioning, relative to its prevalence in the absence of provisioning. (C) Change in prevalence of the ‘interacting’ microparasite (e.g. *E. hungaryensis*) owing to provisioning, relative to its prevalence in the absence of provisioning. In all three figures, the *x-*axis represents the sensitivity of contact rate to provisioning ${(\Theta }_{c})$ and *y-*axis is the sensitivity of host susceptibility to the macroparasite to provisioning ${(\Theta }_{l})$, both expressed relative to the sensitivity of host susceptibility to the microparasites to provisioning. The light-grey regions in the bottom-left of each plot ($\theta_{l},\theta_{c}<\theta_{e}$) show where the provisioning response of host susceptibility to the microparasite is stronger compared with the provisioning response of both host susceptibility to the macroparasite, and contact rate. The horizontal dashed lines show where the provisioning response of host susceptibility to the macroparasite equals that of host susceptibility to the microparasites; the vertical dashed lines show where the provisioning response of host contact rate equals that of susceptibility to the microparasites.

Figure 4 shows contour plots of the predicted changes in mean macroparasite burden (figure 4A) or microparasite infection prevalence (figure 4B,C), from conditions of no provisioning (ρ=0) to high provisioning (ρ=1), for different combinations of $\Theta_{c}$ and $\Theta_{l}$ (sensitivities to provisioning of host contact rate and susceptibility to macroparasite infection, respectively, relative to that of susceptibility to microparasite infection).

Long-term mean macroparasite burden under resource provisioning was predicted to decrease, relative to the value under no provisioning, for approximately half of the combinations of $\Theta_{c}$ and $\Theta_{l}$ explored (figure 4A). Specifically, combinations of low $\Theta_{c}$ (little effect of provisioning on contact rate) and high $\Theta_{l}$ (rapid reduction in host susceptibility to the macroparasite under provisioning) lead to dramatic reductions in mean worm burdens. As the sensitivity of contact rate to provisioning increases $\left(\Theta_{c}>1\right)$, mean worm burden tends to increase considerably under provisioning, but only if the response of worm susceptibility to provisioning is low $\left(\Theta_{l}<∼2\right)$. Indeed, if $\Theta_{l}<1$ (changes in host susceptibility to the macroparasite under provisioning are less than changes in susceptibility to the microparasite) then changes in mean worm burdens under provisioning are relatively insensitive to changes in contact rate beyond $\Theta_{c}>∼2$. When the provisioning response of host susceptibility to microparasites is stronger than that of host susceptibility to the macroparasite $\left(\Theta_{l}<1\right)$, worm mean burden only decreases under provisioning when contact rate is barely affected by provisioning ($\Theta_{c}\sim 0$).

**Figure 5.**
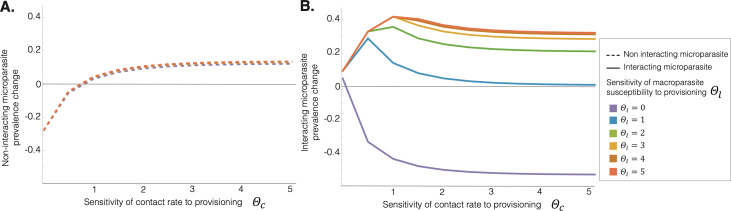
Microparasite prevalence changes induced by provisioning. Figure shows (A) the non-interacting and (B) interacting microparasite prevalence changes owing to sensitivity of contact rate to provisioning ${(\Theta }_{c}$), where each colour represents the sensitivity of host susceptibility to the macroparasite to provisioning ${(\Theta }_{l})$ (i.e. horizontal slices at different points through figure 4B,C, respectively)

For the microparasite that does not interact directly with the macroparasite (e.g. *E. uptoni* in the wood mouse system), the only way co-infection can affect its infection prevalence is through disease-induced host mortality caused by the macroparasite. Thus, prevalence of this microparasite is not substantially affected by changes in the sensitivity of host susceptibility to the macroparasite under provisioning, shown by the vertical lines in figure 4B, particularly for low values of $\Theta_{c}$. Increases of $\Theta_{c}$ generally increase prevalence of the non-interacting microparasite (figure 5A), as contact rate increases with provisioning, facilitating microparasite transmission. However, reducing $\Theta_{l}$ below 1 can reduce prevalence of the non-interacting microparasite, particularly for higher values of $\Theta_{c}$, via the effect of increased macroparasite infections on host mortality.

By contrast, for the microparasite that does interact with the macroparasite (e.g. *E. hungaryensis* in the wood mouse system), when $\Theta_{l}>1$ (i.e. where provisioning sensitivity of host susceptibility to the microparasite is weaker than that of susceptibility to the macroparasite), changes in prevalence of the interacting microparasite follow a hump-shaped relationship with increasing sensitivity of contact rate to provisioning ($\Theta_{c}$), peaking at intermediate values of $\Theta_{c}$ (figure 4C; figure 5B). Since both the microparasites and the macroparasite are assumed to be transmitted through the same route (contact with infective stages, shed from faeces, in the environment), increasing that contact rate initially facilitates infections of both parasite species but, at very high contact rates, the correspondingly high burdens of the macroparasite result in a net reduction in prevalence of the interacting microparasite, via the antagonistic interaction with the co-infecting worms. At low values of $\Theta_{l}$ (particularly when $\Theta_{l}<1$, such that provisioning sensitivity of host susceptibility to the microparasite is weaker than that of susceptibility to the macroparasite), provisioning results in an increasing suppression of microparasite prevalence as the sensitivity of contact rate to provisioning ($\Theta_{c}$) increases (figure 5B). Here, high macroparasite burdens are maintained, owing to the limited change in susceptibility compared to the unprovisioned case, resulting in sustained suppression of the microparasite via the within-host co-infection interaction.

## 4. Discussion

Resource provisioning can have profound consequences for infectious diseases in wildlife [4,5,10]. Several hypotheses have been proposed for why infection prevalence or intensity can increase, decrease or remain unchanged after resource provisioning, including owing to changes in host demography, behaviour and immune responses [4]. However, the potential for differential responses by co-infecting parasites to mediate these effects has so far been ignored, despite the ubiquity of co-infection in natural systems and the known occurrence of interspecific interactions between co-infecting parasites. Here, we use a hybrid microparasite–macroparasite model to show that co-infection interactions can dramatically alter epidemiological responses to provisioning, depending on the balance of sensitivities to provisioning of fundamental mechanisms such as host contact rate and susceptibility to the different parasites.

This work was motivated by known co-infection interactions occurring between the apicomplexan *E. hungaryensis* and the nematode *H. polygyrus* in wood mice (*Apodemus sylvaticus*). This system presents a valuable case study for understanding microparasite–macroparasite co-infection dynamics because (i) both parasites share a common infection site in the gastrointestinal tract of wood mice, and (ii) the presence of the macroparasite (*H. polygyrus*) has been shown to negatively affect the microparasite, *E. hungaryensis*, through experimental studies in both the wild and laboratory [21,22]. Importantly, the wood mouse system also includes another *Eimeria* species, *E. uptoni*, which does not show strong within-host interaction with *H. polygyrus*; this provides a natural control with which to compare and contrast the effects of resource provisioning on closely related ‘interacting’ and ‘non-interacting’ microparasites under co-infection with the macroparasite.

Our modelling of these two scenarios revealed key differences in predicted epidemiological outcomes, which depended on the relative sensitivities to provisioning of host susceptibility to the macroparasite and host contact rates with parasite infective stages in the environment. Previous research has shown that resource provisioning can increase host aggregation and subsequently boost microparasite infection prevalence [7,34]. However, we showed that within-host co-infection interactions can override this effect. Specifically, if host contact rate is relatively unchanged by resource provisioning (low $\Theta_{c}$), both microparasites were predicted to show similar responses to provisioning, generally increasing in prevalence with small increases in contact rate but largely insensitive to changes in host susceptibility to the macroparasite. However, if host contact rates are highly sensitive to provisioning (high $\Theta_{c}$), while prevalence of the non-interacting parasite continues to increase with increasing contact rates under provisioning, prevalence of the interacting parasite declines owing to suppression by the increased macroparasite burdens under high contact rates. As a result, prevalence of the interacting microparasite tends to peak at intermediate sensitivities of contact rate to provisioning (figure 5B).

While we have a good theoretical understanding of how changes in resource provisioning can impact infection dynamics in general, more empirical studies on host–parasite interactions in provisioned and unprovisioned environments are needed. To help bridge between theory and empirical studies, we parameterized our co-infection model with empirical data from the wood mouse system. This parameterization indicated that the baseline recovery time from *E. hungaryensis* infection was estimated to be close to three weeks, which is consistent with previous studies on *Eimeria* infection dynamics [37,38]. However, when co-infected with *H. polygyrus*, this was estimated to be reduced to about one week, supporting the previous evidence of a strong competitive interaction between the pair of parasites. We previously showed via a field supplementation experiment that provisioned wood mice significantly reduced *H. polygyrus* transmission, presumably via reduced host susceptibility to the macroparasite [23]. Based on our results here, we hypothesize that such supplementation would concomitantly increase *E. hungaryensis* prevalence, owing to the release from suppressive effects of *H. polygyrus* co-infection. Moreover, in a resource provisioned co-infection scenario, we predict that an increase in *E. hungaryensis* prevalence may be more responsive to a decrease in the mean burden of *H. polygyrus* than to an increase in the contact rate of *E. hungaryensis*. Future empirical studies will be invaluable for evaluating provisioning results by determining where in the parameter space observed epidemiological responses occur.

Here we did not consider the consequences of demographic changes, such as the host’s carrying capacity, birth and death rates, owing to provisioning. Furthermore, by excluding demographic factors from our model, we do not consider how changes in host population size and structure may interact with contact rates, thereby influencing and potentially increasing transmission rates. Nevertheless, we recognize that when provisioning significantly impacts demography by increasing fecundity and decreasing mortality, it can enhance both pathogen invasion and long-term prevalence [5]. Additionally, we assumed that contact rate increases owing to provisioning [33,34], for example through increased host aggregation around resource patches [8]. Clearly this behaviour would depend on the spatial arrangement of food. If provisioned resources are clustered in space (e.g. such as through a feeder, rubbish dump), it could result in an increase of contact rate between hosts. On the contrary, when provisioned food is dispersed more evenly, contact rates could decrease with increased provisioning. Our previous work on the wood mouse system has shown that within-host co-infection interactions can have spatially structured impacts on transmission dynamics of the respective parasites [39]. We, thus, hypothesize that there may be potentially subtle but important interactions between within-host co-infection interactions and between-host aggregation outcomes dependent on the spatial distribution of resources [40]. We also acknowledge that we did not consider the case in which provisioned food quality is lower compared with the baseline (natural, unprovisioned) scenario, which may reduce potential benefits to hosts via effects on susceptibility to parasites. As such, alternative provisioning scenarios (varying food quality and/or distribution) are likely to alter predicted impacts on co-infection dynamics.

Additionally, several other simplifying assumptions were made in our model to maintain conceptual focus and clarity. First, our model assumes a unidirectional interaction whereby macroparasite burdens affect microparasite recovery, intentionally omitting potential reciprocal interactions that, although biologically plausible and documented in some studies, would greatly increase model complexity. Second, we limited the effects of provisioning to changes in host susceptibility and contact rates, explicitly excluding potential impacts on host mortality or parasite reproduction within hosts. Finally, we assumed that both parasites shared the same contact rate, which is appropriate in the wood mouse system, where both *H. polygyrus* and *Eimeria* spp. are transmitted via ingestion of environmentally shed stages. However, in co-infection systems where parasites differ in transmission mode (e.g. direct contact versus vector-borne), this assumption would not hold and could result in divergent responses to provisioning, ultimately altering co-infection outcomes. While these various additional pathways are biologically relevant in a range of systems and may influence infection dynamics substantially, their inclusion would require detailed system-specific data, reduce general applicability and substantially complicate interpretation. In contrast to Fenton [17], which allowed for bidirectional effects between parasite species—including effects on parasite reproduction and host mortality—our framework intentionally abstracts away from these details to explore general principles of how resource provisioning interacts with co-infection dynamics. Future work could build upon this conceptual foundation by explicitly investigating these complexities in well-characterized empirical systems.

While our model was parameterized using the UK wood mouse system, where we have well-documented evidence of a powerful interaction between two co-infecting parasites*,* the framework is broadly applicable to other co-infection systems where within-host interactions and environmental change shape infection dynamics. For example, in humans, helminths influence responses to co-infecting pathogens such as *Plasmodium falciparum* [41] and human immunodeficiency virus [42], with consequences for disease susceptibility and progression. In the African buffalo (*Syncerus caffer*), helminth infections can impact resistance to *Mycobacterium bovis*, the agent of bovine tuberculosis, through immunomodulation [19,43]. In these systems, and others where co-infection interactions alter within-host infection dynamics, and resource levels may alter host immune responses and/or transmission-relevant contact behaviours, we would expect to see similar outcomes whereby the effects of resource provisioning—through food aid, agriculture or habitat change—are mediated by those within-host co-infection interactions. Clearly, the nature of any such mediation will depend on the direction of co-infection interaction—negative when one parasite suppresses another (as occurring between *H. polygyrus* and *E. hungaryensis* in the wood mouse system) potentially reversing the effects of resource provisioning on the parasite affected by the within-host interaction, or positive when one parasite facilitates the other, potentially exacerbating the effects of resource provisioning. Furthermore, the outcome may also depend on how resources affect host immune responses. Specifically, resource availability can influence susceptibility, resistance and tolerance. Our model assumes provisioning acts by reducing susceptibility, not by altering resistance costs or tolerance. For instance, increased tolerance to macroparasites could permit high helminth burdens to persist and sustain suppression of co-infecting microparasites, while enhanced resistance could reduce macroparasite loads and relieve this suppression. We note that experimental resource supplementation in the wood mouse system has been shown to reduce helminth (*H. polygyrus*) burdens, suggesting a provisioning effect acting on host resistance (reduced susceptibility or increased worm clearance) rather than acting on host tolerance [23]. However, incorporating variation in host defence strategies will be essential for applying this model across systems and improving predictions of how provisioning influences co-infection dynamics.

In summary, we used a hybrid microparasite–macroparasite model to study the role of resource provisioning on co-infection dynamics. We show that when parasites strongly interact within the host, the effects of provisioning on parasite transmission dynamics can be mediated, and potentially overturned, by within-host co-infection interactions. These findings highlight the need for long-term field experiments to disentangle the ecological and epidemiological consequence of resource supplementation in multi-parasite systems.

## Data Availability

Data are available from the Dryad repository [44]. Supplementary material is available online [45].

## References

[1] Bradley CA, Altizer S. 2007 Urbanization and the ecology of wildlife diseases. Trends Ecol. Evol. **22**, 95–102. (10.1016/j.tree.2006.11.001) 17113678 PMC7114918

[2] Gottdenker NL, Streicker DG, Faust CL, Carroll CR. 2014 Anthropogenic land use change and infectious diseases: a review of the evidence. EcoHealth **11**, 619–632. (10.1007/s10393-014-0941-z) 24854248

[3] Oro D, Genovart M, Tavecchia G, Fowler MS, Martínez‐Abraín A. 2013 Ecological and evolutionary implications of food subsidies from humans. Ecol. Lett. **16**, 1501–1514. (10.1111/ele.12187) 24134225

[4] Becker DJ, Streicker DG, Altizer S. 2015 Linking anthropogenic resources to wildlife–pathogen dynamics: a review and meta‐analysis. Ecol. Lett. **18**, 483–495. (10.1111/ele.12428) 25808224 PMC4403965

[5] Becker DJ, Hall RJ. 2014 Too much of a good thing: resource provisioning alters infectious disease dynamics in wildlife. Biol. Lett. **10**, 20140309. (10.1098/rsbl.2014.0309) 25055815 PMC4126624

[6] Goulson D, Whitehorn P, Fowley M. 2012 Influence of urbanisation on the prevalence of protozoan parasites of bumblebees. Ecol. Entomol. **37**, 83–89. (10.1111/j.1365-2311.2011.01334.x)

[7] McCallum H. 2001 How should pathogen transmission be modelled? Trends Ecol. Evol. **16**, 295–300. (10.1016/s0169-5347(01)02144-9) 11369107

[8] Altizer S, Hochachka WM, Dhondt AA. 2004 Seasonal dynamics of mycoplasmal conjunctivitis in eastern North American house finches. J. Anim. Ecol. **73**, 309–322. (10.1111/j.0021-8790.2004.00807.x)

[9] Cypher BL, Frost N. 1999 Condition of San Joaquin kit foxes in urban and exurban habitats. J. Wildl. Manag. **63**, 930.

[10] Erazo D, Pedersen AB, Fenton A. 2022 The predicted impact of resource provisioning on the epidemiological responses of different parasites. J. Anim. Ecol. **91**, 1719–1730. (10.1111/1365-2656.13751) 35643978 PMC9546467

[11] Ezenwa VO. 2016 Helminth–microparasite co‐infection in wildlife: lessons from ruminants, rodents and rabbits. Parasite Immunol. **38**, 527–534. (10.1111/pim.12348) 27426017

[12] Pedersen AB, Fenton A. 2007 Emphasizing the ecology in parasite community ecology. Trends Ecol. Evol. **22**, 133–139. (10.1016/j.tree.2006.11.005) 17137676

[13] Zilio G, Koella JC. 2020 Sequential co‐infections drive parasite competition and the outcome of infection. J. Anim. Ecol. **89**, 2367–2377. (10.1111/1365-2656.13302) 32688437 PMC7589385

[14] Maizels RM, Yazdanbakhsh M. 2003 Immune regulation by helminth parasites: cellular and molecular mechanisms. Nat. Rev. Immunol. **3**, 733–744. (10.1038/nri1183) 12949497

[15] Grencis RK. 2015 Immunity to helminths: resistance, regulation, and susceptibility to gastrointestinal nematodes. Annu. Rev. Immunol. **33**, 201–225. (10.1146/annurev-immunol-032713-120218) 25533702

[16] Graham AL. 2008 Ecological rules governing helminth–microparasite coinfection. Proc. Natl Acad. Sci. USA **105**, 566–570. (10.1073/pnas.0707221105) 18182496 PMC2206576

[17] Fenton A. 2008 Worms and germs: the population dynamic consequences of microparasite-macroparasite co-infection. Parasitology **135**, 1545–1560. (10.1017/s003118200700025x) 18070368

[18] Flynn RJ, Mulcahy G, Welsh M, Cassidy JP, Corbett D, Milligan C, Andersen P, Strain S, McNair J. 2009 Co-infection of cattle with Fasciola hepatica and Mycobacterium bovis - immunological consequences. Transbound. Emerg. Dis. **56**, 269–274. (10.1111/j.1865-1682.2009.01075.x) 19575746

[19] Jolles AE, Ezenwa VO, Etienne RS, Turner WC, Olff H. 2008 Interactions between macroparasites and microparasites drive infection patterns in free-ranging African buffalo. Ecology **89**, 2239–2250. (10.1890/07-0995.1) 18724734

[20] Lello J, Boag B, Fenton A, Stevenson IR, Hudson PJ. 2004 Competition and mutualism among the gut helminths of a mammalian host. Nature **428**, 840–844. (10.1038/nature02490) 15103373

[21] Clerc M, Fenton A, Babayan SA, Pedersen AB. 2019 Parasitic nematodes simultaneously suppress and benefit from coccidian coinfection in their natural mouse host. Parasitology **146**, 1096–1106. (10.1017/s0031182019000192) 30915927 PMC6603796

[22] Knowles SCL, Fenton A, Petchey OL, Jones TR, Barber R, Pedersen AB. 2013 Stability of within-host–parasite communities in a wild mammal system. Proc. R. Soc. B **280**, 20130598. (10.1098/rspb.2013.0598) PMC3673050 23677343

[23] Sweeny AR, Clerc M, Pontifes PA, Venkatesan S, Babayan SA, Pedersen AB. 2021 Supplemented nutrition decreases helminth burden and increases drug efficacy in a natural host–helminth system. Proc. R. Soc. B **288**, 20202722. (10.1098/rspb.2020.2722) PMC7893286 33468010

[24] Knowles SCL, Fenton A, Pedersen AB. 2012 Epidemiology and fitness effects of wood mouse herpesvirus in a natural host population. J. Gen. Virol. **93**, 2447–2456. (10.1099/vir.0.044826-0) 22915692 PMC3542127

[25] Sweeny AR, Albery GF, Venkatesan S, Fenton A, Pedersen AB. 2021 Spatiotemporal variation in drivers of parasitism in a wild wood mouse population. Funct. Ecol. **35**, 1277–1287. (10.1111/1365-2435.13786)

[26] Anderson RM, May RM. 1978 Regulation and stability of host-parasite population interactions: I. Regulatory processes. J. Anim. Ecol. **47**, 219. (10.2307/3933)

[27] Anderson RM, May RM. 2010 Infectious diseases of humans: dynamics and control. Reprinted, p. 757. Oxford, UK: Oxford University Press.

[28] May RM, Anderson RM. 1981 The population dynamics of microparasites and their invertebrate hosts. Phil. Trans. R. Soc. Lond. B **291**, 451–524. 10.1098/rstb.2014.0307 PMC4360116 25750231

[29] Erazo D, Pedersen AB, Gallagher K, Fenton A. 2021 Who acquires infection from whom? Estimating herpesvirus transmission rates between wild rodent host groups. Epidemics **35**, 100451. (10.1016/j.epidem.2021.100451) 33761448

[30] Graham AL, Allen JE, Read AF. 2005 Evolutionary causes and consequences of immunopathology. Annu. Rev. Ecol. Evol. Syst. **36**, 373–397. (10.1146/annurev.ecolsys.36.102003.152622)

[31] Råberg L, Graham AL, Read AF. 2009 Decomposing health: tolerance and resistance to parasites in animals. Phil. Trans. R. Soc. B **364**, 37–49. (10.1098/rstb.2008.0184) 18926971 PMC2666700

[32] McCarville J, Ayres J. 2018 Disease tolerance: concept and mechanisms. Curr. Opin. Immunol. **50**, 88–93. (10.1016/j.coi.2017.12.003) 29253642 PMC5884632

[33] Plowright RK, Foley P, Field HE, Dobson AP, Foley JE, Eby P, Daszak P. 2011 Urban habituation, ecological connectivity and epidemic dampening: the emergence of hendra virus from flying foxes (Pteropus spp.). Proc. R. Soc. B **278**, 3703–3712. (10.1098/rspb.2011.0522) PMC3203503 21561971

[34] Wright AN, Gompper ME. 2005 Altered parasite assemblages in raccoons in response to manipulated resource availability. Oecologia **144**, 148–156. (10.1007/s00442-005-0018-3) 15891856

[35] Eley RM, Strum SC, Muchemi G, Reid GDF. 1989 Nutrition, body condition, activity patterns, and parasitism of free-ranging troops of olive baboons (Papio anubis) in Kenya. Am. J. Primatol. **18**, 209–219. (10.1002/ajp.1350180304) 31964035

[36] French SS, Fokidis HB, Moore MC. 2008 Variation in stress and innate immunity in the tree lizard (Urosaurus ornatus) across an urban–rural gradient. J. Comp. Physiol. B **178**, 997–1005. (10.1007/s00360-008-0290-8) 18594834 PMC2774757

[37] Higgs S, Nowell F. 1988 Laboratory studies with clones of Eimeria hungaryensis, a parasite of the wood mouse Apodemus sylvaticus. Parasitology **97**, 213–220. (10.1017/s0031182000058418) 3200603

[38] Nowell F, Higgs S. 1989 Eimeria species infecting wood mice (genus Apodemus) and the transfer of two species to Mus musculus. Parasitology **98**, 329–336. (10.1017/s0031182000061394) 2528109

[39] Keegan SP, Pedersen AB, Fenton A. 2024 The impact of within-host coinfection interactions on between-host parasite transmission dynamics varies with spatial scale. Proc. R. Soc. B **291**, 20240103. (10.1098/rspb.2024.0103) PMC11021925 38628126

[40] Creech TG, Cross PC, Scurlock BM, Maichak EJ, Rogerson JD, Henningsen JC, Creel S. 2012 Effects of low‐density feeding on elk–fetus contact rates on Wyoming feedgrounds. J. Wildl. Manag. **76**, 877–886. (10.1002/jwmg.331)

[41] Pullan RL, Kabatereine NB, Bukirwa H, Staedke SG, Brooker S. 2011 Heterogeneities and consequences of plasmodium species and hookworm coinfection: a population based study in Uganda. J. Infect. Dis. **203**, 406–417. (10.1093/infdis/jiq063) 21187338 PMC3038339

[42] Walson JL, Herrin BR, John-Stewart G. 2009 Deworming helminth co-infected individuals for delaying HIV disease progression. Cochrane Database Syst. Rev. CD006419. (10.1002/14651858.cd006419.pub3) 19588389 PMC2871762

[43] Ezenwa VO, Jolles AE. 2015 Opposite effects of anthelmintic treatment on microbial infection at individual versus population scales. Science **347**, 175–177. (10.1126/science.1261714) 25574023

[44] Erazo D, Sweeny AR, Pedersen AB, Fenton A. 2025 Data from: Parasite responses to resource provisioning can be altered by within-host co-infection interactions. Dryad Digital Repository. (10.5061/dryad.msbcc2g72)41187922

[45] Erazo D, Sweeny AR, Pedersen A, Fenton A. 2025 Supplementary material from: Parasite responses to resource provisioning can be altered by within-host co-infection interactions. Figshare. (10.6084/m9.figshare.c.8065732)41187922

